# CRISPR and the Future of Cardiac Disease Therapy: A New Genetic Frontier

**DOI:** 10.3390/ijms27083641

**Published:** 2026-04-19

**Authors:** Sem Sterckel, Imelda Lizeth Chávez Martínez, Verena Schwach

**Affiliations:** Applied Stem Cell Technologies Group, Department of BioEngineering Technologies, Cardiovascular Health Technology Centre, Techmed Centre, University of Twente, 7522 Enschede, The Netherlands; s.a.sterckel@utwente.nl (S.S.); imeldalizeth2011@hotmail.com (I.L.C.M.)

**Keywords:** CRISPR-Cas, genome editing, cardiovascular disease, disease modeling, gene therapy, CRISPR screens, delivery systems, clinical translation

## Abstract

CRISPR technologies are transforming cardiovascular therapy development by creating an increasingly seamless pipeline from potential target discovery to clinical translation. What began as a genome-editing tool has evolved into a versatile platform that enables researchers to precisely interrogate and modulate cardiac biology with tools such as base- and prime-editors, and CRISPR inhibition and activation. In this review, we follow the use of CRISPR across the stages of biomedical research through to bench-to-bedside application. This review begins by addressing how genome-wide and focused CRISPR screens discover developmental regulators, disease drivers, and drug-response pathways, making the first steps in identifying therapeutic targets. We then explore how CRISPR engineering creates progressively more relevant disease model systems to validate mechanisms of disease and test interventions, helping bridge the translational gaps between the lab and the clinic. Finally, we consider how CRISPR technologies are beginning to enter cardiovascular clinical trials, while highlighting the key challenges that still limit this translation. By linking the latest advances of modern CRISPR platforms to the stages of therapeutic development, this review highlights how CRISPR technology is reshaping the pipeline from molecular insight to clinical innovation in cardiac disease.

## 1. Introduction

Few breakthroughs in modern biology have reshaped the scientific landscape as profoundly as clustered regularly interspaced short palindromic repeats (CRISPR) and the associated Cas proteins. Originally discovered as an adaptive immune mechanism in bacteria, CRISPR has rapidly risen from a molecular curiosity to one of the most versatile and powerful genetic tools ever developed, leading to the recognition of the Nobel Prize in chemistry [1]. With the ability to target virtually any genomic sequence using a programmable RNA guide, CRISPR enables precise, efficient, and accessible manipulation of DNA, an advance that has transformed research across nearly every domain of life sciences [2].

Yet the often-told story of CRISPR-Cas simple genome cutting is far richer. Over the past decade, the technology has evolved into a diverse toolkit: from refined approaches to homology directed repair (HDR), to next-generation systems such as nickases, base editors, and prime editors that achieve nucleotide-level changes without double-strand breaks, and CRISPR activation and inhibition systems which can manipulate expression levels. These advances have been paralleled by rapid improvements in delivery technologies, expanding the range of tissues and disease contexts in which CRISPR can be deployed.

In this review, we explore how these diverse CRISPR technologies are accelerating the discovery and development of therapies for cardiac disease. We examine genome-wide CRISPR screens as engines for identifying developmental regulators, disease drivers, and therapeutic targets. We review how CRISPR-enabled disease models, ranging from isogenic human pluripotent stem cell (hPSC) in vitro platforms to in vivo animal studies, advance mechanistic insight and therapeutic testing. Subsequently, we discuss strategies to increase the translational relevance of these disease models in order to minimize high attrition rates of newly developed drugs. Finally, we survey the current landscape of cardiovascular clinical trials employing CRISPR technologies, highlighting where momentum is building and where major translational challenges remain.

Together, these perspectives illustrate how CRISPR is reshaping both our understanding of cardiac biology and the future of cardiac therapeutics.

## 2. Gene Manipulation Toolkit

The CRISPR-Cas system is composed of the Cas protein and the single guide RNA (sgRNA). The sgRNA comprises the fusion of two crucial components, namely a CRISPR RNA (crRNA) and a trans-activating crRNA (tracrRNA). While the crRNA carries the complementary sequence to the target DNA, the tracrRNA serves as a scaffold, binding to the crRNA and facilitating the recruitment of the Cas protein to the target DNA. Together, these components ensure precise guidance of the Cas protein to the target DNA sequence [2]. Upon binding to the target site, the Cas endonuclease initiates DNA cleavage, inducing a double-stranded break (DSB) at the designated locus [2]. This step allows for the modification, deletion, or insertion of DNA sequences to facilitate gene editing.

CRISPR-Cas technologies predominantly leverage the Streptococcus pyogenes (SpCas9) variant [3]. Nonetheless, a diverse array of Cas9 orthologues has emerged as viable options for genome editing [4,5,6]. These orthologues exhibit notable disparities in size [7], protospacer adjacent motif (PAM) sequences [6,8], editing efficiency [9], and specificity [6,9]. The specificity of Cas9 activity crucially hinges on the recognition of the PAM, a specific DNA sequence adjacent to the target site. For instance, the commonly utilized SpCas9 system requires an NGG sequence as the PAM, where N denotes any nucleotide. To expand the spectrum of targetable regions, researchers have engineered Cas9 variants with modified PAM requirements [3,10]. Beyond Cas9, recent advancements have introduced new RNA-guided endonucleases, such as Fanzor, which open additional possibilities in genome editing. These eukaryotic endonucleases offer unique advantages, including their smaller size, making them ideal for delivery, and enhanced specificity, reducing off-target effects compared to Cas9 variants [11].

**Figure 1:**
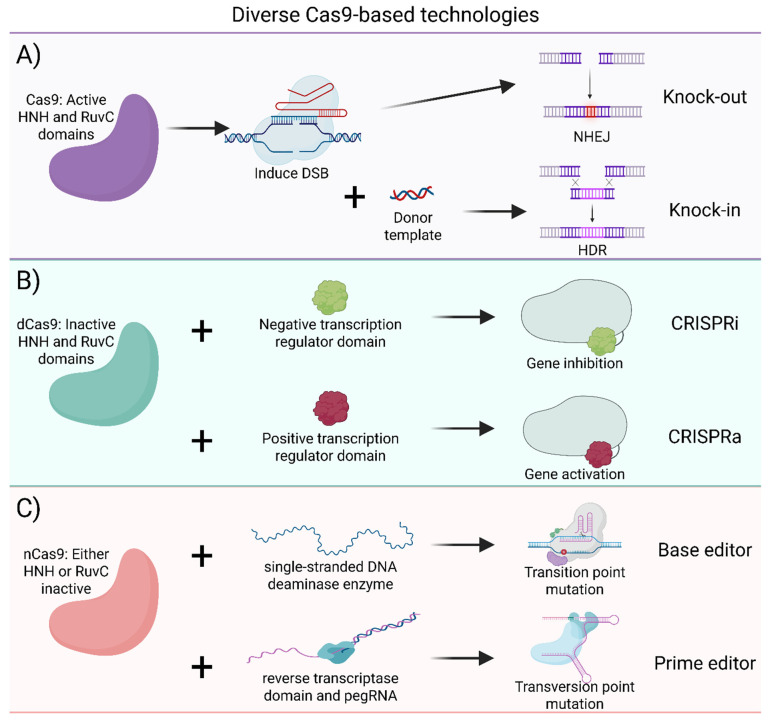
Cas9-based technologies. (**A**) A Cas9 enzyme with active HNH and RuvC domains induces a DBS which can result in a knock-out, or a knock-in if a donor template is present. (**B**) A dCas9 protein with inactive HNH and RuvC domains can be coupled to negative or positive transcription regulator domains to inhibit or activate a gene, respectively. (**C**) An nCas9 enzyme with either an inactive HNH domain or inactive RuvC domain can give rise to a base editor or a prime editor [17].

Following the creation of a DSB at the target genomic locus, cellular DNA repair pathways come into play to resolve the lesion and facilitate genomic modifications. Two principal repair pathways, non-homologous end joining (NHEJ) and HDR, dictate the outcome of gene editing (Figure 1A). NHEJ operates by directly ligating the broken ends together, without necessitating perfect alignment [6]. NHEJ is characterized by its rapid and versatile nature, as it doesn’t require a template for repair. However, NHEJ often leads to random substitutions, insertions and deletions (indels), rendering it error-prone. Nonetheless, this pathway can be harnessed for gene function disruption, commonly referred to as knock-outs [12]. In contrast to NHEJ, during HDR, the cell utilizes a homologous DNA sequence as a template to accurately repair the damaged DNA, thereby ensuring genomic integrity [11]. In the context of CRISPR-Cas technology, HDR provides a mechanism for precise genetic alteration. By utilizing either double-stranded DNA (dsDNA) or single-stranded DNA oligonucleotides (ssODNs) as templates, HDR facilitates applications, including gene knock-ins, deletions, or corrections [13]. Despite the advantages of HDR, eukaryotic cells typically exhibit a preference for the error-prone NHEJ pathway, posing challenges for CRISPR-Cas-mediated knock-ins. To address this issue, various chemical, physical, and genetic strategies have been devised to either impede NHEJ or enhance HDR [14,15,16].

### 2.1. Enhancing HDR Efficiency: Strategies and Limitations

Improving the efficiency of HDR remains a central challenge in precise genome editing, particularly for therapeutic applications. One widely explored approach involves the use of small molecules (See Table 1) that either inhibit key NHEJ components (e.g., DNA-PKcs, 53BP1) or activate HDR-promoting factors such as RAD51 [18,19]. Among small molecules, RS-1 (a RAD51 stimulator) enhances strand invasion, while Scr7, NU7441, and M3814 inhibit DNA ligase IV or DNA-PKcs, key components of NHEJ. Mirin and B02 target MRE11 and RAD51, respectively, modulating the DNA damage response to favor HDR [18]. Histone deacetylase inhibitors like trichostatin A and valproic acid have also been shown to increase chromatin accessibility, facilitating template integration [19]. Cell cycle synchronization, such as arresting cells in the S or G2 phase using agents like nocodazole or thymidine, where HDR is naturally more active, can significantly boost editing precision [18,19]. Additionally, modifying the design of donor DNA templates, such as using ssODNs, asymmetric homology arms, and chemical modifications (e.g., phosphorothioate bonds), has been shown to improve integration efficiency [18]. Cas9 fusion proteins, such as Cas9-RAD52 or Cas9-Geminin, have been engineered to recruit HDR machinery or restrict activity to HDR-permissive cell cycle phases [20,21]. Another simple, yet highly effective, method to enhance HDR is the post-transfection ‘cold-shock’ at 32 °C for 24–48 h, which increases editing efficiencies two- to ten-fold in hPSCs [22]. More recently, prime editing and CRISPR-associated transposases (CASTs) have emerged as alternatives that bypass the need for double-strand breaks altogether, offering precise insertions with reduced reliance on HDR [23].

Collectively, these strategies reflect a growing toolkit aimed at refining genome editing outcomes for both research and therapeutic applications. Despite these advances, several challenges and limitations remain. Many HDR-enhancing molecules exhibit cell-type specificity or cytotoxicity, limiting their generalizability [18,19]. HDR remains inefficient in non-dividing cells, such as neurons or cardiomyocytes, which are critical targets for many genetic therapies [18]. Moreover, manipulating DNA repair pathways can lead to genomic instability, off-target effects, or unintended large deletions [18]. In addition, timing the delivery of CRISPR components and donor templates to coincide with the optimal cell cycle phase is technically demanding and remains an active area of investigation [19,23]. As the field evolves, combining multiple strategies, including chemical, genetic, and structural, may offer the most robust path forward for safe and efficient genome editing.

### 2.2. Unlocking the Potential of Cas9: From Precision Nicks to Prime Editing

Cas9 comprises two distinct nuclease domains: HNH and RuvC. The HNH domain is responsible for cleaving the target DNA strand, while the RuvC domain cleaves the PAM-containing non-target DNA strand [2]. By selectively inactivating one or both nuclease domains, the enzymatic activity of Cas9 can be modulated to alter its cleavage properties (See Figure 1).

When either one of the nuclease domains in Cas9 is inactivated, it gives rise to a Cas9 nickase (nCas9). Unlike the wild-type Cas9, the nCas9 cleaves only one DNA strand, inducing a single-strand break (SSB) rather than a DSB, making it less prone to off-target effects [16]. This additional safety makes nickases a preferred choice for clinical applications [24]. In contrast, when both nuclease domains of Cas9 are rendered inactive, it results in the generation of a catalytically dead Cas9 variant (dCas9). Unlike its active counterpart, dCas9 lacks DNA cleavage capability and functions primarily by binding to the target DNA sequence with high specificity [25]. dCas9 can be harnessed for precise transcriptional regulation of target genes through two distinct strategies: CRISPR interference (CRISPRi) and CRISPR activation (CRISPRa). CRISPRi and CRISPRa offer transient regulation of gene expression, typically lasting a few days in primary cells, instead of permanently altering the DNA [26] (Figure 1B). In CRISPRi, dCas9 is coupled with negative transcription regulation domains, such as KRAB, SID, and SRDX, leading to the suppression of gene expression [27,28]. Conversely, CRISPRa employs dCas9 fused with positive transcriptional regulation domains, such as VPR, Suntag, or SAM [24], to promote the upregulation of target genes [29,30]. It is also worth noting an inverse correlation between basal activity and relative activation achieved, meaning that CRISPRa has a bigger impact on the upregulation of factors with otherwise low activity [31]. This underscores the importance of selecting a potent transcriptional regulator when aiming to upregulate a highly expressed gene. For instance, ACTC1 upregulation with VP64 results in only a 3.5-fold increase over basal levels, whereas VPR boosts expression by 330-fold [32].

The utility of catalytically inactive Cas9 variants extends beyond transcriptional modulation. By fusing these programmable DNA-binding proteins to different effector domains, entirely new classes of precision tools can be created. A key example of this is the development of base editors, a groundbreaking class of genome editing tools designed for precise chemical modification of DNA sequences at the nucleotide level. Comprising either an nCas9 or a dCas9 fused with a single-stranded DNA deaminase enzyme, base editors offer the capability to induce specific nucleotide changes without the need for DSBs or external DNA templates. There are two main categories of base editors: cytosine base editors (CBEs) and adenine base editors (ABEs) [33,34]. CBEs facilitate the conversion of C-G base pairs to T-A base pairs, while ABEs catalyze the reverse reaction, converting A-T base pairs to G-C base pairs. Collectively, these base editors enable the installation of the four transition point mutations: C to T, A to G, T to C, and G to A, providing researchers with precise control over nucleotide alterations within the genome. Like DSB-based CRISPR technologies, various Cas9 orthologs can be used to engineer base editors, enabling access to different targets due to diverse PAM requirements and allowing for smaller base editing complexes [35]. Despite their remarkable precision, traditional base editors have limitations. They cannot introduce transversion point mutations (C-G to A-T, C-G to G-C, T-A to A-T, and T-A to G-C), small insertions, or deletions. Prime editing, introduced by Anzalone et al. in 2019 [36], addresses these limitations by utilizing a fusion of nCas9 with an engineered reverse transcriptase domain, along with a prime-editing guide RNA (pegRNA). This innovative system allows for a broader range of mutations, including transversions and small indels, while maintaining high editing precision and efficiency [36].

Altogether, the diversification of CRISPR systems highlights the maturation of genome engineering into a precise and multifunctional toolkit. With these capabilities established, the effectiveness of any CRISPR-based technology increasingly hinges on the ability to deliver these components to specific tissues and cell types, determining the utility in both research and therapy.

## 3. The Art of Getting CRISPR in: Delivery

**Figure 2:**
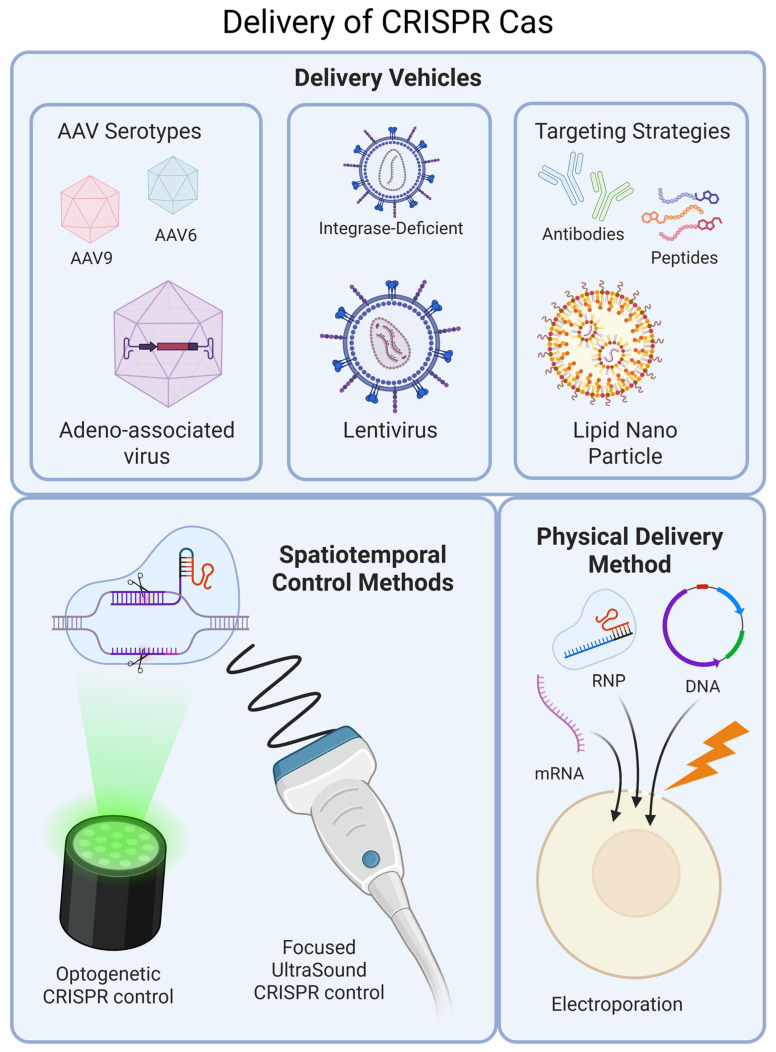
Overview of CRISPR Cas delivery techniques, including delivery vehicles and optimization strategies, spatiotemporal control methods, and a physical delivery method [37].

In both in vitro and in vivo models, efficient delivery of CRISPR-Cas components to target cells is essential for potent therapeutic applications and achieving stronger research insights. Delivery systems must not only protect these cargos from degradation but also facilitate their efficient uptake by target cells while minimizing off-target effects, immune responses, and cytotoxicity. In this chapter, we explore the diverse array of delivery methods, including viral, non-viral, and physical approaches (See Figure 2).

### 3.1. CRISPR Building Blocks: gRNA and Cas Protein Formats

The CRISPR-Cas system consists of a Cas nuclease guided by a gRNA to a specific DNA target, and can be delivered in several formats, including DNA, mRNA, and pre-formed ribonucleoprotein (RNP) complexes, each with distinct advantages and limitations. DNA-based delivery is straightforward to produce and relies on the host cell’s transcriptional and translational machinery to generate sustained Cas expression [38]. However, prolonged nuclease activity increases the risk of off-target editing, and DNA delivery carries additional concerns such as random genomic integration, insertional mutagenesis, and immune responses, which limit its clinical applicability [38].

In contrast, mRNA and RNP delivery formats avoid genomic integration and enable transient Cas activity, thereby reducing long-term off-target risks [39]. mRNA delivery allows rapid Cas expression without permanent genetic modification, but its relatively low stability, dependence on host translation, and susceptibility to innate immune activation can reduce editing efficiency [39]. RNP delivery offers the most precise temporal control, as the Cas protein is immediately active upon delivery and rapidly degraded, resulting in high editing efficiency, improved specificity, and minimal toxicity [40]. Nonetheless, RNPs present challenges for in vivo delivery due to limited cellular uptake and short half-lives, and may still induce immune responses because of the bacterial origin of the Cas proteins [40].

### 3.2. Viral Vectors: The Golden Standard for In Vivo Editing

Viral vectors remain a cornerstone for CRISPR-Cas9 delivery in mouse models of genetic cardiomyopathies due to their high efficiency and stable expression [41]. Among these, Adeno-Associated Virus (AAV) has been the most widely used vector, particularly the AAV9 serotype, which exhibits strong cardiac tropism [42]. A showcase application of AAV-mediated CRISPR-Cas9 was demonstrated in Lamin A/C (*LMNA*)-mutant mouse models of dilated cardiomyopathy (DCM). In a recent study, researchers delivered a therapeutic transgene via AAV9 to restore normal nuclear-cytoskeletal interactions, leading to preserved ejection fraction and extended lifespan [43]. However, the small cargo capacity of AAV (~4.7 kb) poses a limitation, often necessitating dual-vector approaches to separately deliver Cas9 and guide RNAs [41]. Beyond AAV, lentiviral vectors are frequently used to deliver CRISPR guide RNA libraries for functional genomics studies in cardiovascular research such as CRISPR screens in hPSC-derived cardiomyocytes [44,45]. Lentiviral vectors ensure long-term expression through integrating into the genome [46]. To counter possible concerns about insertional mutagenesis, integrase-deficient lentiviral vectors (IDLVs) have been developed, allowing for transient expression [47]. While this development shows a positive prospect for in vivo editing with lentiviruses, they are still considered mostly suitable for in vitro studies and ex vivo cell therapy.

### 3.3. Nanoparticles: A Non-Viral Alternative for Systemic Gene Editing

Lipid nanoparticles (LNPs) are trending as a non-viral alternative for CRISPR-Cas9 delivery, offering transient gene editing with reduced immunogenicity [48]. Their versatility allows encapsulation of Cas9 mRNA and sgRNA, enabling efficient in vivo genome editing while minimizing off-target effects. LNPs have shown promise in atherosclerosis models, particularly in targeting Proprotein Convertase Subtilisin/Kexin Type 9 (*PCSK9*) for cholesterol regulation [49]. Interest in LNPs for the delivery of CRISPR Cas elements is growing, with several clinical trials currently running [50,51,52]. However, while LNPs have been highly successful in liver-targeted delivery of CRISPR elements (as seen in the editing rate of 31% in extracted liver cells when targeting Angiopoietin-like 3 (*ANGPTL3*) gene using LNPs [53]), their efficiency in cardiomyocyte transfection remains a challenge. To improve targeting specificity, recent efforts have focused on surface modifications, such as conjugating antibodies or peptides that enhance uptake by cardiac tissues [48].

Beyond LNPs, advances in nanotechnology have introduced gold nanoparticles (GNPs), silica nanoparticles (SiNPs), and exosome-based delivery systems as novel CRISPR-Cas9 carriers [54]. Gold nanoparticles conjugated with Cas9 RNPs offer an additional strategy for transient gene editing with low toxicity, demonstrated in preclinical models of Duchenne muscular dystrophy [55]. These nanoparticle-based approaches show great potential and delivery efficiency in vitro, but remain in early-stage development, and the lack of cardiac specificity remains a limitation for cardiac therapy in vivo [54].

### 3.4. Physical Delivery Methods: High-Efficiency Gene Editing In Vitro

For in vitro gene editing, physical delivery methods like electroporation and nucleofection are widely used in hPSCs due to their high efficiency in transiently delivering RNP complexes or sgRNA into cells [56,57]. These features make the delivery method ideal for disease model creation or CRISPR screens. Recent studies using electroporation achieved >90% genome-editing efficiency via HDR in hPSC without significant loss of viability [58,59]. While highly effective for preclinical research, electroporation is fundamentally unsuitable for most cardiovascular therapeutic applications. Beyond its lack of tissue specificity and impracticality for whole organ gene editing, in vivo electroporation induces direct tissue injury, inflammation, and fibrosis, and results in highly heterogeneous and spatially confined genome editing. These limitations are highlighted in dystrophin restoration studies in mdx mice, where Cas9-mediated editing was limited to directly electroporated muscle regions [60]. Such localized and invasive delivery is particularly problematic in cardiac tissue, where structural disruption, electrical heterogeneity, and mosaic editing may exacerbate arrhythmia risk and impair synchronized contraction. Together, these drawbacks confine in vivo electroporation to proof-of-concept studies and limit its use as a clinically translatable strategy for cardiac gene editing.

### 3.5. Spatiotemporal Control: A Non-Invasive Leap Toward Precision Gene Editing

The integration of CRISPR-Cas systems with non-invasive triggers enables fine spatiotemporal control of genome editing. A pioneering platform from Peter Yingxiao Wang’s team uses focused ultrasound (FUS) to generate localized hyperthermia that activates heat shock promoters placed upstream of CRISPR effectors, thereby switching CRISPRa/CRISPRi/CRISPR tools (FUS CRISPRa, FUS CRISPRe, FUS CRISPR) on only at targeted tissue sites [61]. This strategy delivers reversible, repeatable, and highly localized editing. Complementing acoustic control, optogenetic CRISPR systems achieve similar external regulation using light [62]. For example, Pulgarin D. V. et al. (2025) highlight BLU VIPR, where blue light-responsive Vivid Photoreceptor Domain (VVD) photoreceptors are fused to dCas9 effectors [63]. Illumination induces VVD dimerization and assembly of the active CRISPR complex only within the illuminated region, enabling reversible, high-resolution transcriptional modulation without chemical inducers. Conceptually, the same light or ultrasound responsive design can be modularized to gate different steps in CRISPR therapeutics, for example, by directly controlling Cas protein activity or localization, tuning gRNA availability/stability, or activating the delivery vehicle itself. These optogenetic and FUS CRISPR systems could enhance clinical adaptability of CRISPR systems by enabling spatiotemporal on-demand activation in hard-to-reach organs such as heart tissue, improving specificity, safety, and therapeutic feasibility, where current delivery systems struggle.

### 3.6. Remaining Barriers: Cardiac Efficiency and Specificity

While delivery strategies for CRISPR have been extensively reviewed elsewhere, their performance and safety constraints are of particular importance in cardiovascular applications. In the heart, current viral and non-viral delivery vehicles remain limited by safety, biodistribution, and efficacy challenges, despite substantial technological advances. Both AAV-based vectors and LNP formulations typically require high systemic doses to achieve meaningful editing in the heart, leading to hepatic accumulation, inflammation, and dose-dependent toxicity [48,64]. In contrast, therapies focusing on liver-targeted delivery, such as *PCSK9* and *ANGPTL3* editing, can benefit from the hepatic accumulation and thus require lower dosages for adequate effects [49,53]. Although repeated LNP administration could theoretically improve cardiac editing efficiency, this strategy further increases the risk of cumulative vehicle-mediated off-target effects and systemic toxicity [48].

A further challenge is mosaicism, as only a subset of cardiomyocytes tends to receive CRISPR cargo with current technologies. Partial editing can be beneficial in disorders such as Duchenne muscular dystrophy, where even modest dystrophin restoration improves muscle function [65,66]. However, in arrhythmogenic and conduction-related cardiomyopathies, uneven editing may disrupt electrical synchrony and increase arrhythmia risk, representing a unique off-target consequence at the tissue level. Taken together, these considerations underscore the need to independently and critically evaluate vehicle-derived risks when developing CRISPR-based therapies for cardiovascular disease.

## 4. CRISPR in Cardiovascular Therapeutic Research: From Concept to Clinic

With the molecular foundations and delivery principles of CRISPR established, their impact becomes most evident in the diverse ways they are now applied across biological and biomedical research. In the following sections, we examine how CRISPR enables gene discovery through genome screens, supports mechanistic insight through engineered disease models, and drives emerging therapeutic approaches currently advancing toward clinical use in the cardiovascular field.

### 4.1. CRISPR Screens: Mapping Developmental Regulators and Therapeutic Targets for Future Cardiac Gene Editing

CRISPR screening technologies are a foundational technique for developing future cardiovascular therapeutics through the systematic and high-throughput interrogation of genes involved in development, disease mechanisms, and drug responses. These interrogations can be performed using a pooled screening approach, where diverse gRNAs are simultaneously delivered and, through either positive selection (enriching desired phenotypes) or negative selection (depleting defective populations), essential genes can be identified. In early developmental studies, pooled CRISPR screens combined with FACS-based selection allow precise isolation of lineage-specific populations, as demonstrated in a study using MESP1/ISL1 hPSC lines, which ultimately showed that Zinc Family Member 2 (ZIC2), previously known as a neurogenesis regulator, plays an equally vital role in cardiac progenitor formation [67]. Similarly, a myosin heavy chain 6 (MYH6)-GFP (green fluorescence protein) reporter system revealed NF2 as a key regulator of cardiomyocyte specification through YAP signaling [68]. In addition, a recent genome-wide CRISPR knockout screen identified BRD4 as a key regulator of human cardiomyocyte differentiation [69]. Together, these studies highlight how pooled CRISPR screening approaches enable competition between genetic variants in a shared cellular environment, preserving physiologically relevant interactions, while simultaneously uncovering previously unrecognized regulatory nodes governing cardiac lineage commitment.

CRISPR screening has also provided crucial insights into cardiovascular disease mechanisms, such as hypercholesterolemia. One study developed an innovative screening approach using lipoprotein-rich and depleted conditions to identify both shared and condition-specific regulators of LDL uptake [70]. Another study discovered that transgelin modulates LDL receptor internalization through actin cytoskeleton remodeling, providing a potential explanation for cases of familial hypercholesterolemia without Low-Density Lipoprotein Receptor (*LDLR*) mutations [71].

In the context of cardiotoxicity, a genome-wide knockout screen in hPSC-derived cardiomyocytes identified the human-specific transporters SLCO1A2 and SLCO1B3 as critical mediators of Doxorubicin toxicity [45]. In contrast to such unbiased discovery, focused CRISPR libraries concentrate on biologically or therapeutically relevant gene sets, enabling interrogation with fewer cells and lower costs. Complementing the genome-wide findings, a bidirectional CRISPRi/a focused screen combined with a small-molecule pipeline identified CA12 as a previously unrecognized mediator of Doxorubicin-induced cardiotoxicity [72]. Genetic suppression of *CA12* improved cell survival and contractile function, and a CA12 inhibitor mitigated cardiotoxicity in follow-up experiments. Together, these results show how CRISPRi/a screening can uncover metabolic drivers of cardiotoxicity and directly nominate therapeutic targets.

Beyond in vitro applications, an in vivo focused screening approach employed AAV9-delivered gRNAs to target transcriptional regulators in mouse hearts [73]. This work identified the RNF20/40 complex as a key epigenetic modulator of cardiac maturation through its regulation of H2B ubiquitination. By uncovering in vivo regulators of cardiomyocyte maturation, this study provides mechanistic insights that are directly relevant to cardiac regenerative medicine, where promoting the maturation and functional integration of newly generated cardiomyocytes remains a central challenge.

As the application of CRISPR screening technologies in cardiovascular research continues to evolve, a major limitation arises. Because most CRISPR screens are performed in simplified in vitro systems that do not fully recapitulate the structure, physiology, or environmental cues of native heart tissue, their translational value can be limited, positioning them primarily as tools for fundamental discovery rather than direct therapeutic prediction. Nevertheless, by identifying developmental regulators, disease drivers, drug response genes, and emerging therapeutic targets, these screens serve as the earliest step in the “concept to clinic” trajectory of CRISPR-enabled cardiovascular medicine.

### 4.2. Disease Modeling and Therapeutic Development

After CRISPR screens identify potential regulators of development, disease pathways, or therapeutic responses, these candidates must be validated in relevant model systems that capture the appropriate cellular and genetic context. Human-induced pluripotent stem cells (hiPSCs) are one of the key players in this next step because they can be differentiated into cardiomyocytes and other cardiovascular lineages while preserving the genetic background of the donor [74,75]. This allows researchers to model disease mechanisms in a way that overcomes the species-specific limitations of animal systems [76,77]. An example of the translational limitations of animal models can be found in cardiac drug safety assessment. The effects on the human ether-a-go-go-related gene (*hERG*) are not fully captured in conventional models, leading to an unpredicted risk in prolongation of the QT interval for human patients [76]. Importantly, combining hiPSCs with precise CRISPR editing enables the creation of isogenic lines, in which disease-associated variants are introduced or corrected in otherwise identical genomes, providing a direct comparison to attribute phenotypes to specific genetic changes [78]. Despite the remaining challenge of cellular immaturity, hiPSC based models offer a scalable, patient specific, and mechanistically informative model in addition to animal models that anchor the transition from early gene discovery to robust cardiovascular disease modeling [74].

A clear example showing the hiPSC modeling approach comes from Limpitikul, B. et al. (2017), who developed a hiPSC-derived cardiomyocyte model of CALM2-D130G–mediated long QT syndrome (LQTS) [79]. These cells exhibited prolonged action potential duration and disrupted calcium handling, mimicking the clinical phenotype. The authors then applied CRISPR interference to suppress mutant CALM2 expression, which normalized electrophysiological properties, highlighting how disease models can be both mechanistic and therapeutic platforms.

As introduced earlier, isogenic CRISPR-edited hiPSC lines provide controlled comparative models to isolate the effects of individual genetic variants. Building on this concept, Dutton, L. et al. (2024) used CRISPR-Cas9 to introduce the R820W mutation in the *MYBPC3* gene in hiPSCs, modeling hypertrophic cardiomyopathy [80]. Mutant cells showed cellular hypertrophy and impaired relaxation kinetics compared to the isogenic control, illustrating how single mutations can drive complex phenotypes. This combination of mutant and isogenic cell lines can be used to pinpoint key disease mechanisms and potential therapeutic strategies in subsequent research.

Similarly, transgenic approaches have enabled CRISPR-based editing in vivo to develop disease models. Carroll, K. J. et al. (2016) developed a Myh6 promoter-driven spCas9 transgenic mouse model, allowing for cardiac-specific gene deletion upon AAV9-mediated sgRNA delivery [81]. Delivering Myh6 targeting sgRNA resulted in severe cardiomyopathy, demonstrating cardiac-specific in vivo gene editing. Similarly, Sano, S. et al. (2018) used lentiviral delivery of Cas9 and sgRNA to inactivate *TET2* and *DNMT3a* mutations in lineage-negative bone marrow cells of mice [82]. The study highlighted differences in their effects on hematopoietic expansion and inflammatory gene expression, leading to heart failure.

Beyond modeling, CRISPR can be used therapeutically to reverse disease phenotypes by correcting mutations or restoring functional protein expression. Duchenne muscular dystrophy (DMD) is a prime example, with numerous studies demonstrating functional recovery via CRISPR using varying delivery strategies. By utilizing a single-cut strategy with self-complementary AAV (scAAV) and single-stranded AAV (ssAAV) the dystrophin open reading frame could be restored, leading to improved cardiac function and skeletal muscle strength [66]. Using different AAV serotypes, AAV8 and AAV9, exon 23 could be deleted in mdx mice, which led to partial dystrophin recovery and enhanced muscle force [65,83]. Similar delivery strategies using different cargo, namely saCas9 (from Staphylococcus aureus) and multiplex CRISPR approaches, were employed, showing successful dystrophin restoration in muscle stem cells and myoblasts [84,85]. In patient-derived models, plasmid electroporation was used as a delivery mechanism to subsequently perform exon skipping, frameshifting, and exon knock-in strategies in patient hiPSCs, effectively restoring dystrophin expression [86]. In addition to disease models using nucleofection, two gRNA-Cas9 complexes were delivered to DMD fibroblasts to delete exon 51 and correct the reading frame, which were then transdifferentiated into myotubes with recovered dystrophin expression [87]. And lastly using transplantation of patient-derived muscle stem cells, which were edited using spCas9 and Cas12a, into mice, restored dystrophin expression [88]. These studies show the large variation of different CRISPR technologies and delivery methods which are employed for researching potential therapeutic strategies for cardiac diseases.

Currently, CRISPR technologies show great promise in therapeutic strategies targeting monogenic cardiovascular diseases, such as mutated *LMNA*-based diseases like Hutchinson-Gilford progeria syndrome (HGPS). Beyret, E. et al. (2019) employed a two-cut gRNA strategy delivered via AAV9 to target *LMNA* mutations in a HGPS mouse model [89]. This intervention reduced progerin expression, improved activity levels, and extended lifespan. Koblan, L. W. et al. (2021) used an ABE to revert the *LMNA* c.1824C>T mutation, reducing progerin levels and preserving vascular smooth muscle cells, leading to improved survival and cardiovascular health [43].

Especially, the field of vascular health has been taking large steps in improving diseased phenotypes in in vivo disease models using CRISPR technology. Not through targeting vasculature but by using liver-specific LNP delivery of an ABE as mRNA and gRNA targeting *ANGPTL3* in LDLR-deficient mice reduced blood ANGPTL3 protein levels [90]. A different study using the same strategy showed the long-term downregulation of ANGPTL3 protein, low-density lipoprotein cholesterol, and triglyceride levels, parameters that are frequently monitored because of their association with cardiovascular and metabolic risk [53]. This highlights the potential of CRISPR technologies as a therapy for hypercholesterolemia regulation.

Recent advances in precision genome editing have enabled novel therapeutic strategies for non-genetic cardiovascular disease, exemplified by the work of Eric N. Olson and colleagues. In a humanized mouse model of ischemia/reperfusion injury, the team employed ABEs delivered via a myotropic AAV vector to modify oxidation-sensitive methionine residues in CaMKIIδ [91]. This intervention significantly improved cardiac function, reduced fibrosis, and enhanced exercise capacity after myocardial infarction. In a complementary study, the group ablated the autophosphorylation site of CaMKIIδ, a modification known to drive chronic kinase activation in heart failure [92]. This edit conferred robust protection against pressure overload-induced heart failure and arrhythmias, both in vivo and in hiPSC-derived cardiomyocytes. Collectively, these studies highlight the therapeutic potential of precise, non-disruptive base editing of *CaMKIIδ* as a broadly applicable strategy for treating myocardial infarction and heart failure. This opens the possibility that CRISPR could be extended to therapeutic applications in non-genetic diseases when applied in a highly targeted and well-designed manner.

Although recent studies highlight meaningful progress in applying CRISPR technologies to cardiovascular disease models, a fundamental limitation remains: in vivo editing efficiency in the heart is still relatively low, even under optimized experimental conditions. Systemic AAV delivery has shown approximately 15–30% editing in whole-heart DNA in some models, which was sufficient to yield measurable phenotypic improvement [43,66]. Direct intramyocardial AAV injection can raise these numbers locally, with up to 37% editing in the anterior cardiac wall [91]. While these results are technically impressive for a densely structured, post-mitotic organ, they nonetheless reflect partial and mosaic editing rather than widespread genetic modification in cardiomyocytes.

Importantly, whole-tissue editing percentages can overestimate the degree of editing within cardiomyocytes themselves, as cardiac tissue contains numerous non-cardiomyocyte cell types, including fibroblasts, endothelial cells, and immune cells, that are more easily transduced. For many cardiac diseases, particularly those involving conduction abnormalities or contractile dysfunction, therapeutic benefit likely requires substantially higher editing rates in cardiomyocytes than those currently achievable. Taken together, these findings emphasize that limited cardiac editing efficiency remains a major bottleneck for CRISPR-based therapies, underscoring the need for next-generation delivery systems with far greater cardiomyocyte specificity and penetration.

#### Bridging the Gap Between Lab and Clinic

Despite the sophistication of modern CRISPR-based disease models, a persistent challenge remains: the translational gap between laboratory success and clinical efficacy. Many therapeutics that robustly rescue phenotypes in reductionist systems ultimately fail to progress toward clinical trials because the models used capture only a fraction of human physiological complexity [93,94]. Simple 2D in vitro cultures, though invaluable for rapidly validating gene edits or establishing mechanistic causality, fall short in recapitulating the integrated electrical, mechanical, and metabolic demands of the human heart [95,96,97]. These limitations can mask off-target physiological effects, alter drug responsiveness, or obscure emergent tissue-level behaviors that only arise in multicellular, mechanically loaded environments. As a result, conclusions drawn from early in vitro rescue experiments may overestimate the therapeutic potential of CRISPR interventions when deployed in vivo.

Advanced bioengineered systems such as engineered heart tissues and organ-on-chip platforms offer a critical intermediate step by introducing 3D architecture, biomechanical load, vascular-mimicking perfusion, and more physiologic electrical integration [95,96,97]. These systems bridge the divide between simple monolayers and whole animals, enabling researchers to evaluate how precise genome edits influence contractility, conduction, arrhythmia susceptibility, and long-term tissue remodeling, phenotypes that are invisible in conventional cell culture. Complementary in vivo models, meanwhile, remain essential for assessing whole-organ dynamics, immune interactions, systemic delivery barriers, and off-target consequences across organs [94]. They are especially important for stress-testing delivery modalities such as AAVs or LNPs, which are, in the field of CRISPR-based therapeutics for cardiovascular disease, a main bottleneck [7]. Yet, animal models can still fail to reproduce human-specific mechanisms, particularly in genetic diseases where subtle differences in regulatory networks or protein isoforms shift disease trajectories [94]. And since genetic diseases are expected to be the most potent targeted diseases for CRISPR-based therapeutics, it seems essential to test on a holistic panel of models [43,98]. Taken together, these layers of complexity underscore the importance of using a diversified model ecosystem, spanning 2D assays, 3D tissues, organ-on-chip systems, and in vivo platforms, to more accurately predict which CRISPR-based therapies are truly poised for successful clinical translation.

### 4.3. Clinical Applications

As CRISPR technologies advance from early discovery and preclinical modeling to real-world application, the emerging clinical trials in cardiovascular medicine provide an essential opportunity to observe how genome editing performs in human patients (See Table 2). Although still limited in number and scope, these first in human studies offer valuable insights into the practical challenges and design considerations that will shape future therapeutic development. By examining these early efforts, we can begin to understand not only where CRISPR-based interventions are currently being deployed, but also what gaps remain in translating genome editing into effective treatments for the full spectrum of cardiovascular diseases.

One of the most advanced cardiovascular clinical applications of in vivo CRISPR/Cas9 gene editing is in the treatment of transthyretin amyloidosis (ATTR), a progressive and often fatal disease caused by the accumulation of misfolded transthyretin (TTR) protein in tissues [99,100]. This accumulation can lead to cardiomyopathy, heart failure, and peripheral neuropathy, depending on the clinical subtype. ATTR exists in both hereditary and wild-type forms. NTLA-2001, developed by Intellia Therapeutics, delivers CRISPR/Cas9 via LNPs to hepatocytes, the primary site of TTR production. Following a one-time intravenous infusion, Cas9 mRNA and a TTR-specific sgRNA are taken up by the liver, where Cas9 introduces double-strand breaks that are repaired through NHEJ, permanently disrupting TTR expression. Preclinical studies demonstrated durable TTR knockdown after a single dose, and early clinical data from the Phase I trial (NCT04601051) showed that NTLA-2001 achieved up to 93% reduction in serum TTR levels in patients with hereditary ATTR amyloidosis with polyneuropathy and ATTR-related cardiomyopathy [52,100]. This represents the first-ever clinical evidence of in vivo CRISPR gene editing in human cardiovascular disease, marking a significant milestone in the field of genomic medicine. This approach not only offers a potential one-time treatment for a disease that currently requires lifelong therapy, but also sets a precedent for future in vivo gene editing strategies targeting liver-expressed genes. Building on this momentum, Intellia has advanced a next-generation TTR editing therapeutic, exiguran ziclumeran (Nex-Z), into a global Phase 3 clinical trial [101].

A major advancement in the clinical application of CRISPR for cardiovascular disease is exemplified by in vivo CRISPR/Cas9 gene editing therapies, CTX310 (ACTRN12623000809639) and CTX320 (ACTRN12623001095651), developed by CRISPR Therapeutics [51,102]. Both utilize LNP delivery of CRISPR/Cas9 components, Cas9 mRNA and sgRNA, to target genes in the liver. CTX310 is designed to induce NHEJ and subsequently permanently disrupt the *ANGPTL3* gene, a key regulator of lipid metabolism. Loss-of-function mutations are associated with reduced triglycerides, LDL cholesterol, and a lower risk of atherosclerotic disease. Following promising preclinical results demonstrating significant reductions in ANGPTL3 protein and serum lipid levels, a Phase 1 clinical trial was launched in 2023 to evaluate CTX310 as a one-time treatment for familial hypercholesterolemia and atherosclerosis. Results already show a reduced ANGPTL3 protein reduction in serum of 89% [103]. In parallel, CTX320 targets the Lipoprotein(a) (*LPA*) gene, which encodes lipoprotein(a), an independent and genetically determined risk factor for atherosclerotic disease and is inadequately addressed by current therapies. Preclinical studies in non-human primates showed durable LPA reductions after a single dose of CTX320, prompting the initiation of a Phase 1 trial to assess its safety, tolerability, and efficacy in humans. Together, CTX310 and CTX320 show how single-dose liver-targeted gene editing may offer long-term therapeutic benefit for common, non-monogenic lipid disorders, which traditionally require lifelong management.

In addition to nuclease-based therapies, base editing approaches are also advancing into clinical evaluation for cardiovascular disease. Developed by Verve Therapeutics, both VERVE 101 (NCT05398029) and VERVE 102 (NCT06164730) utilize ABEs delivered as mRNA encoding the base editor together with a guide RNA packaged within lipid nanoparticles, enabling precise A-T to G-C substitutions in the *PCSK9* gene [50,104]. This single-nucleotide edit disrupts PCSK9 function, reducing LDL receptor degradation and yielding sustained lowering of circulating LDL cholesterol levels. VERVE-101 delivered via first-generation LNPs demonstrated potent LDL C reduction in early participants [105]. However, the trial was halted after a patient receiving a higher dose experienced abnormalities in liver enzymes and platelets, findings attributed to the LNP formulation rather than the base editor itself. This highlighted a key translational barrier, namely that the delivery chemistry, not editing precision, can determine clinical viability. VERVE 102, now in Phase 1b evaluation, uses a redesigned GalNAc LNP system to improve hepatocyte targeting and mitigate the safety concerns encountered with VERVE 101 [50].

Current clinical efforts in CRISPR-based cardiovascular therapy overwhelmingly focus on liver-directed genome editing delivered via LNPs, aiming to improve vascular health or mitigate cardiomyopathic effects driven by systemic protein dysregulation. Beyond the leading programs described above, several additional first-in-human studies, including VERVE-201 [106], YOLT-101 [107], ART001 [108], ART002 [109], and CS-121 [110,111], further reinforce this trend. Although these trials vary in editing mechanism and therapeutic target, they all follow the same fundamental strategy: The leveraging of hepatocyte-directed LNP delivery to edit drivers of lipid metabolism or circulating pathogenic proteins, thereby achieving systemic cardiovascular benefit through a single in vivo intervention.

**Table 2:** Summary of CRISPR-based Clinical Trials for Cardiovascular Diseases as of March 2026.

Trial Name	Target Gene	Condition	Delivery	Mechanism	Phase	Developer
NTLA-2001 [52] (NCT04601051)/ Nexiguran ziclumeran [101] (NCT06128629)	*TTR*	ATTR causing cardiomyopathy	LNP IV infusion Cas9 mRNA and gRNA	DSB to induce a knockout of TTR	Phase 3 ongoing	Intellia Therapeutics
CTX310 [51] (ACTRN12623000809639)	*ANGPTL3*	Familial hypercholesterolemia and ASCVD	LNP IV infusion Cas9 mRNA and gRNA	DSB to induce a knockout of ANGPTL3 to reduce LDL cholesterol and triglycerides	Phase 1 ongoing	CRISPR Therapeutics
CTX320 [102] (ACTRN12623001095651)	*LPA*	ASCVD	LNP IV infusion Cas9 mRNA and gRNA	DSB to induce a knockout of LPA	Phase 1 ongoing	CRISPR Therapeutics
VERVE-101 [104] (NCT05398029)	*PCSK9*	HeFH	LNP IV infusion ABE mRNA and gRNA	Base editing to disrupt PCSK9 expression to reduce LDL cholesterol	Completed	Verve Therapeutics
VERVE-102 [50] (NCT06164730)	*PCSK9*	HeFH	GalNAc-LNP IV infusion ABE mRNA and gRNA	Base editing to disrupt PCSK9 expression to reduce LDL cholesterol	Phase 1 ongoing	Verve Therapeutics
VERVE-201 [106] (NCT06451770)	*ANGPTL3*	ASCVD	GalNAc-LNP IV infusion ABE mRNA and gRNA	Base edit to disrupt ANGPTL3 expression to reduce LDL cholesterol and triglycerides	Phase 1 ongoing	Verve Therapeutics
YOLT-101 [107] (NCT06458010)	*PCSK9*	HeFH	GalNAc-LNP IV infusion ABE mRNA and gRNA	Base editing to disrupt PCSK9 expression to reduce LDL cholesterol	IIT Early Phase 1 ongoing	YolTech Therapeutics
ART001 [108] (ChiCTR2400081216)	*TTR*	ATTR causing cardiomyopathy	LNP IV infusion Cas9 mRNA and gRNA	DSB to induce a knockout of TTR	Phase 2 ongoing	AccurEdit Therapeutics
ART002 [109] (NCT07353398)	*PCSK9*	HeFH	LNP IV infusion Cas9 mRNA and gRNA	DSB to induce a knockout of PCSK9 to reduce LDL cholesterol	IIT Early Phase 1 soon to open	AccurEdit Therapeutics
CS-121 [110,111] (NCT07176923)/(NCT07371767)	*APOC3*	Hyperchylomicronemia	LNP IV infusion transformer base editor mRNA and gRNA	Base editing to disrupt APOC3 expression	Early Phase 1 ongoing	Correct- Sequence Therapeutics

Abbreviations: ATTR: Transthyretin amyloidosis, LNP IV infusion: Intravenous LNP infusion, DSB: Double-stranded break, ASCVD: atherosclerotic cardiovascular disease, HeFH: Heterozygous familial hypercholesterolemia, IIT: Investigator-Initiated Trial.

Despite major advances in mechanistic studies and an improving landscape of preclinical cardiac models, no clinical trials have yet attempted in vivo editing of cardiomyocytes. This gap is mainly caused by the translational barrier of efficient, safe, and targeted delivery of CRISPR cargo to heart tissue in humans. Unlike the liver, where LNP uptake is naturally efficient, cardiac tissue lacks an equivalent entry route, making myocardial gene editing far more challenging [48]. Looking forward, advances improving overall delivery methods, including ligand-decorated LNPs, next-generation AAV capsids with enhanced cardiac tropism, and emerging non-viral nanoparticle platforms, offer promising avenues to overcome the heart’s natural resistance to nucleic acid uptake [41,47,48,54]. Complementary progress in spatiotemporal control systems, such as ultrasound-responsive promoters or optogenetic CRISPR assembly, may further enable precise activation of genome editors only within defined cardiac regions [61,62]. Together, these emerging technologies highlight a path toward future trials that move beyond liver-restricted editing and begin to address direct, in situ correction of genetic heart disease.

## 5. Conclusions

CRISPR-Cas technologies continue to evolve at an exceptional pace, transforming both fundamental biology and translational biomedical research. Beyond classical genome editing via DSB repaired by HDR or NHEJ, the field has rapidly expanded to include programmable transcriptional control systems such as CRISPR interference and activation, as well as precision editing tools like base editors and prime editors that enable single-nucleotide or small-template modifications without introducing DSBs. Parallel engineering of new Cas variants, whether orthologs with distinct PAM requirements or compact proteins optimized for delivery, has further broadened the genomic reach and therapeutic potential of CRISPR-based systems.

As an application, high-throughput CRISPR screens have significantly accelerated biological discovery by enabling systematic interrogation of gene function at scale. These approaches have deepened our understanding of developmental regulators, essential genes, disease drivers, drug response pathways, and emerging therapeutic targets. The ability to map such complex gene–phenotype relationships in an unbiased manner has made CRISPR screening an essential tool for both fundamental and translational science.

In disease modeling, the synergy between CRISPR engineering and either human hiPSCs technology or animal models has been of unmistakable value. Researchers can generate isogenic pairs differing only in specific genetic alterations, enabling precise dissection of disease mechanisms with high control. These platforms also provide robust testbeds for evaluating CRISPR-based therapeutic interventions, with many studies demonstrating effective correction of pathogenic mutations or modulation of disease pathways directly in patient-derived or isogenic models. The emergence of more complex systems, including 3D-engineered tissues, organoids, and organ-on-chip devices, promises to further strengthen this pipeline. By pairing isogenic hPSC lines with physiologically relevant microenvironments, these advanced models can improve the prediction of therapeutic efficacy and reduce the high attrition rates typically seen when transitioning to clinical trials.

Clinically, CRISPR therapeutics within the cardiovascular field are emerging, with current trials primarily targeting liver tissue using LNP-delivered editors to modulate circulating proteins relevant to vascular health or cardiomyopathy. This bias in current clinical trials highlights a major translational bottleneck: the difficulty of achieving efficient and safe delivery of CRISPR agents to cardiac tissue. Overcoming this challenge, whether through adaptations on nanoparticles, improved AAV tropism, or spatiotemporally precise modalities such as ultrasound- or optogenetically controlled CRISPR systems, will be essential for unlocking CRISPR-based therapies for cardiac diseases. Overall, the impact of CRISPR in cardiovascular medicine is likely to depend less on further refinement of genome editing itself and more on advances in tissue-specific delivery and safety. If these limitations are not addressed, even well-validated and highly precise genetic interventions are unlikely to progress beyond early proof-of-concept studies. Shifting the focus from editing efficiency to effective and controlled delivery may therefore be key to the continued development of CRISPR-based cardiovascular therapies.

## Figures and Tables

**Table 1:** Homology-Directed Repair Enhancement Molecules in Genome Editing.

Mechanism/Category	Representative Molecules	Effect on HDR	Notes/Limitations
RAD51 Modulators	RS-1 (stimulator), B02 (inhibitor)	RS-1: enhances strand invasion and promotes HDR. B02: modulates HDR suppression.	RS-1: Cell-type specificity. B02: Used to study HDR suppression.
NHEJ Inhibitors (DNA-PKcs/Ligase IV inhibition)	Scr7 (Ligase IV inhibitor), NU7441, M3814, VX-984 (DNA-PKcs inhibitors)	Suppresses NHEJ.	Potential off-target effects.
MRE11 Inhibitor	Mirin	Modulates DNA damage response.	Used to study HDR suppression.
Histone Deacetylase (HDAC) Inhibitors	Trichostatin A, Valproic Acid	Increases chromatin accessibility.	Potential toxicity and broad epigenetic effects.
β3-Adrenergic Receptor Agonist	L755507	Increases HDR efficiency.	Variable efficacy

## Data Availability

No new data were created or analyzed in this study. Data sharing is not applicable to this article.

## Abbreviations

The following abbreviations are used in this manuscript:

AAV	Adeno-Associated Virus
AAV9	Adeno-Associated Virus serotype 9
ABE	Adenine Base Editor
ANGPTL3	Angiopoietin-like 3
ATTR	Transthyretin Amyloidosis
CASTs	CRISPR-Associated Transposases
CBE	Cytosine Base Editor
CRISPR	Clustered Regularly Interspaced Short Palindromic Repeats
CRISPRa	CRISPR Activation
CRISPRi	CRISPR Interference
crRNA	CRISPR RNA
dCas9	Dead Cas9
DCM	Dilated Cardiomyopathy
DMD	Duchenne Muscular Dystrophy
dsDNA	Double-Stranded DNA
DSB	Double-Strand Break
FUS	Focused Ultrasound
GFP	Green Fluorescent Protein
GNPs	Gold Nanoparticles
HDR	Homology-Directed Repair
hERG	Human Ether-à-go-go–Related Gene
hPSC	Human Pluripotent Stem Cell
hiPSC	Human-Induced Pluripotent Stem Cell
HGPS	Hutchinson–Gilford Progeria Syndrome
IDLV	Integrase-Deficient Lentiviral Vector
LDLR	Low-Density Lipoprotein Receptor
LPA	Lipoprotein(a)
LMNA	Lamin A/C
LNP	Lipid Nanoparticle
LQTS	Long QT Syndrome
mRNA	Messenger RNA
MYH6	Myosin Heavy Chain 6
nCas9	Cas9 Nickase
NHEJ	Non-Homologous End Joining
PAM	Protospacer Adjacent Motif
PCSK9	Proprotein Convertase Subtilisin/Kexin Type 9
pegRNA	Prime Editing Guide RNA
RNP	Ribonucleoprotein
scAAV	Self-Complementary Adeno-Associated Virus
sgRNA	Single Guide RNA
siNPs	Silica Nanoparticles
ssAAV	Single-Stranded Adeno-Associated Virus
SSB	Single-Strand Break
ssODNs	Single-Stranded Oligodeoxynucleotides
tracrRNA	Trans-Activating CRISPR RNA
VVD	Vivid Photoreceptor Domain
ZIC2	Zinc Finger Protein of the Cerebellum 2
